# Single nucleotide polymorphisms, gene expression and evaluation of immunological, antioxidant, and pathological parameters associated with bacterial pneumonia in Barki sheep

**DOI:** 10.1186/s13620-025-00296-1

**Published:** 2025-04-12

**Authors:** Ahmed El Sayed, Amani Hafez, Ahmed Ateya, Asmaa Darwish, Amin Tahoun

**Affiliations:** 1 Department of Animal Health and Poultry, Animal and Poultry Production Division, Desert Research Center (DRC), Cairo, Egypt; 2 Department of Development of Animal, of Veterinary Medicine, Mansoura University, Mansoura, Egypt; 3 Department of Animal Medicine, Faculty of Veterinary Medicine, Kafrelshkh University, Kafr El Sheikh, 33516 Egypt; 4 Departments of Veterinary Clinical Sciences, Faculty of Veterinary Medicine, Jordan University of Science and Technology, Irbid, 22110 Jordan

**Keywords:** Antioxidant, Barki sheep, Gene expression, Immunity, Nucleotide sequence variants, Pneumonia

## Abstract

**Background:**

In sheep, pneumonia is a major concern because of its high morbidity, mortality, and economic impact. It results from various infectious agents, including bacteria, viruses, and environmental stressors, that weaken the immune system.

**Objective:**

The objective of this study was to monitor nucleotide sequence variations, gene expression, and serum biomarkers of inflammation and oxidative stress in sheep with pneumonia. Additionally, this study aimed to identify various bacterial strains and virulent gene combinations in pneumonic sheep, as confirmed by PCR.

**Methodology:**

The enrolled animals were categorized as follows: 50 apparently healthy ewes, considered the control group, and 150 infected ewes with pneumonia. The infected ewes included 100 sporadic cases from the Center for Sustainable Development of Matrouh Resources, Desert Research Center, Matrouh, Egypt, and 50 ewes from the slaughterhouse, all exhibiting respiratory symptoms such as coughing, serous to mucopurulent nasal discharge, fever, and abnormal lung sounds. Blood samples were collected to assess various biochemical parameters, detect SNPs, and analyse the expression of specific immunological and antioxidant-related genes. Nasopharyngeal and lung swabs were taken from the affected ewes for bacteriological analysis, and lung samples were collected for histological examination.

**Results:**

Phenotypic characterization and identification revealed the presence of *Klebsiella pneumoniae*,* Pasteurella multocida*,* Mannheimia haemolytica*,* Pseudomonas* spp., *Mycoplasma*,* Streptococcus*, and *Escherichia coli*, with frequencies of 40%, 28.6%, 34%, 18%, 44%, 29.3%, and 20%, respectively. Additionally, virulence genes for *Klebsiella pneumoniae*, *iutA* and *fimH*, were detected at rates of 39% and 68%, respectively, whereas the *toxA* gene for *Pseudomonas* spp. was present in 59.2% of the cases. Nucleotide sequence variations in immunity- and antioxidant-related genes were observed between healthy and pneumonic ewes. The genes encoding *IL-1α*,* IL1B*,* IL6*,* TNF-α*,* LFA-1*,* CR2*,* IL17*,* IL13*,* DEFB123*,* SCART1*,* ICAM1*,* NOS*, and *HMOX1* were significantly upregulated in pneumonia-affected ewes compared with resistant ewes. Conversely, the genes encoding *IL10*,* SOD1*,* CAT*,* GPX1*, and *NQO1* were downregulated. Further analysis of the serum profile revealed a significant (*P* < 0.05) increase in IL-1α, IL-1β, IL-6, TNF-α, NO and MDA along with a significant (*P* < 0.05) decrease in the serum levels of C3, C4, CAT, GPx, GR and IL-10 in diseased ewes compared with healthy ewes. Histopathological examination revealed that the infected sheep exhibited broncho-interstitial pneumonia and purulent to fibrino-purulent bronchopneumonia.

**Conclusions:**

This study revealed the significant presence of various pathogens and virulence factors in infected sheep, along with distinct immunological and antioxidant gene expression patterns. The altered serum profile and gene regulation in pneumonia-affected ewes underscore the complex immune response and potential biomarkers for disease susceptibility and resistance.

## Introduction

The Barki sheep breed, named after the Libyan province of Barka, is native to Egypt’s northwest coastal region and is essential to the local population’s way of life [1]. With a population of 470,000, accounting for 11% of Egypt’s total sheep. This breed is spread across a vast area, from eastern Libya to the western part of Alexandria, Egypt. Barki sheep exhibit remarkable tolerance to extreme temperatures, limited forage, and heat stress and play a prominent role in this region [2]. Basic information about the body conformation and productivity of Barki ewes is available [3].

The health of livestock populations worldwide is severely threatened by bacterial and viral respiratory diseases [4, 5]. In addition, predisposing management factors such as stressful environmental conditions such as overcrowding, transportation, abrupt climate changes, inadequate ventilation, and poor nutrition typically result in lung infections that cause significant losses. Respiratory disorders in sheep frequently develop as a result of immunosuppressive stress conditions, which may lead to secondary infections or primary infections caused by bacterial and/or viral agents [6–9].

Pneumonia typically arises from an inflammatory response in the bronchioles and alveoli of the lungs caused by substances that lead to the solidification of lung tissue. According to [10], lung defense mechanisms become weakened, and disease can develop when a threshold level of pathogens, host vulnerability, and nonspecific defense responses is reached. Pneumonia is responsible for 40–70% of sheep losses and affects 30–40% of flocks. The economic costs associated with sheep pneumonia include reduced growth rates, milk production, increased morbidity and mortality, carcass condemnation, and the expense of vaccination and chemotherapy programs [11, 12]. In Egypt, the highest incidence of sheep pneumonia typically occurs during autumn and early winter, as well as in spring and early summer. This is due to sudden temperature shifts from warm to cold. Additionally, dust particles that carry infections deep into the respiratory system are more prevalent in spring and autumn [13, 14]. To maintain production and meet the demand for meat and milk, eliminating or mitigating the impact of diseases that affect sheep is crucial [15].

Several bacteria have been isolated from the infected lungs of sheep, including *Staphylococcus aureus*, *Escherichia coli*, *Klebsiella pneumoniae*, *Bacillus* species, *Streptococcus pyogenes*, *Corynebacterium* species, *Pasteurella* species, and *Arcanobacterium pyogenes* [16, 17].

A wide range of clinical issues in small ruminants, especially respiratory conditions, are linked to various *Mycoplasma species*. These problems result in significant economic losses, particularly in African countries [18]. These bacteria are highly specific and require particular substrates to grow in controlled environments [19, 20]. Species such as *Mycoplasma arginini*, *Mycoplasma ovipneumoniae*, and *Mycoplasma agalactiae* have been identified and isolated in Egypt [21]. *E. coli*, a common member of the Enterobacteriaceae family, is frequently observed in respiratory infections in sheep, where it can be isolated from both pneumonia-affected lungs and nasal swabs [22, 23].

*Pasteurella multocida*, an opportunistic bacterium, is one of the most serious pathogens affecting domestic ruminants, causing acute pneumonia outbreaks, particularly in animals under stress from factors such as weaning, transport, environmental changes, or primary respiratory infections [24, 25]. In normally benign nasopharyngeal commensals, these bacteria can become harmful when an animal’s immune defenses are weakened [26]. *Pseudomonas aeruginosa* causes a variety of diseases in sheep and goats, but respiratory illnesses, especially pneumonia, are the main cause of concern because they lead to significant mortality and increased financial losses [27]. *Klebsiella pneumoniae* carries the fimH gene, a virulence factor believed to increase bacterial adherence to epithelial cells. Another important virulence gene, iutA, increases the expression of other pathogenic genes, contributing to greater harm [28].

Polymerase chain reaction (PCR) is the most efficient and rapid method for identifying microbial pathogens in clinical samples. PCR is especially useful for detecting pathogens that are difficult to culture or require extended cultivation periods [29]. This method allows for faster diagnosis, enabling quicker treatment and reducing the use of antimicrobial drugs, which helps lower both costs and side effects [30].

Oxidative stress often occurs when there is an imbalance between antioxidants and oxidants, such as free radicals or reactive oxygen species. However, animal bodies can regulate excess free radicals through defense mechanisms [31, 32]. Catalases and other antioxidant enzymes are crucial to these processes [33]. Cytokines, a class of soluble proteins, regulate cellular and tissue activity. They have a short half-life, are produced locally in response to stimuli, and can function in an endocrine, exocrine, or autocrine manner [34]. Sheep that have acute pneumonia are at risk for organ failure and death due to a severe proinflammatory state that increases the release of inflammatory cytokines [35].

Marker-assisted selection (MAS) is a genomic technique that helps in the development of disease-resistant animals by selecting for alleles at the DNA level rather than relying on a phenotypically expressed disease state. This approach enhances selection accuracy without being influenced by environmental factors, making it a valuable tool for improving the disease resistance of livestock [36]. Molecular genetic techniques also hold promise for partially addressing the shortcomings of conventional methods used by animal breeders in their efforts to improve the health of their animals [37]. The exposure of animals to infections is one of the main obstacles to using selection for disease-resistant animals. Since there is no guarantee that animals bred for reproduction are treated humanely, it is impractical to reject this practice. Many beneficial single nucleotide polymorphisms (SNPs) have been described as a result of extensive efforts to identify DNA markers for sickness resistance [38]. This study aimed to investigate the bacterial species and virulence gene combinations isolated from sheep with respiratory symptoms and to verify them via PCR. This work also explored the histological and macroscopic abnormalities in the affected lungs. Additionally, this study assessed how proinflammatory cytokines, oxidative stress biomarkers, and genetic polymorphisms could be used to identify pneumonia in Barki sheep.

## Materials and methods

### Animals and study design

A total of 200 Barki ewes, aged 3 to 5 years (mean ± SD: 3.8 ± 0.6) and weighing between 28 and 43 kg (mean ± SD: 35 ± 5), were included in this study. On the basis of a thorough clinical examination following the methodology outlined by [39], the ewes were divided into two groups according to assessments of heart, lung, and rumen health, along with other vital signs. Group 1 (*n* = 50), comprising clinically healthy ewes, was assigned to the healthy control group (HCG), which presented a normal body temperature, pulse, respiration rate, clear eyes, absence of nasal or lacrimal discharge, normal posture, and appetite. The pneumonic group (PG) consisted of 150 ewes displaying clinical signs of respiratory disease, including fever, abdominal breathing, mucopurulent nasal discharge, weakness, appetite loss, and abnormal lung sounds (crackles and wheezes). This group included 50 ewes from a slaughterhouse and 100 sporadic cases from the Center for Sustainable Development of Matrouh Resources, Desert Research Center, Matrouh, Egypt.

The ewes were housed in semiopen shaded pens and provided a daily diet consisting of 750 g of concentrated feed mixture (CFM) and 750 g of alfalfa hay per head, which was offered twice daily with unrestricted water access. When available, they also had access to natural pasture, including grass, berseem, darawa, and green herbage. The CFM composition included wheat bran (240 kg), soybean meal (230 kg), corn (530 kg), sodium chloride (5 kg), calcium carbonate (10 kg), premix (1 kg), Netro-Nill (0.5 kg), and Fylax (0.5 kg).

### Sampling

#### Blood sampling

Ten milliliters of blood were collected from each animal via jugular venipuncture. The samples were divided into plain tubes (without anticoagulant) for serum collection and EDTA tubes for whole blood collection. The samples were immediately chilled on crushed ice and transported to the laboratory. Whole blood was used for DNA and RNA extraction, while serum samples were obtained via centrifugation in plain blood tubes at 3000 rpm for 15 min, aliquoted, and stored at −20 °C for biochemical analysis of oxidative and energetic stress markers.

#### Nasopharyngeal swabs

A total of 100 nasopharyngeal swabs were collected. Each animal was identified, restrained, and secured by an assistant. After the nasal exterior was disinfected with 70% alcohol, a sterile cotton-tipped swab was inserted into the nostril and rotated against the nasal cavity wall. Swabs were placed in sterile test tubes containing 10 mL of tryptone soy broth, brain heart infusion broth, and PPLO broth and stored in an icebox for transport to the Microbiology Department, Desert Research Center, Matrouh. Sampling was conducted on the basis of evidence of recurrent pneumonia symptoms.

#### Emergencies slaughtered (lung swabs)

Fifty lungs were subjected to standard postmortem meat inspection. Suspicious lung surfaces were incised with a sterile scalpel, and inner surfaces were sampled via sterile swabs. The samples were transported to the Microbiology Department following the same protocol as the nasal swabs [40]. Affected lung tissues and associated lymph nodes were collected in 10% neutral-buffered formalin (NBF) for histopathology, while frozen tissues were stored at −20 °C for further analysis.

#### Pathological investigation

Tissue samples (~ 1 cm³) were fixed in 10% NBF for histological analysis. The samples were dehydrated in graded ethanol, embedded in paraffin, sectioned at 5 μm thickness, stained with hematoxylin and eosin, and examined under a standard light microscope [41].

#### Bacterial isolation

Samples from affected lung lesions were aseptically collected and transported to the Bacteriology Department. The pneumonic lung surface was cauterized with a heated spatula before the inner tissue was cultured on blood agar with 5% sheep blood, Baird‒Parker agar, mannitol salt agar, Pseudomonas cetrimide agar, oxacillin resistance screening agar basal medium (ORSAB) [42], and MacConkey agar. The plates were incubated aerobically at 37 °C for 24–48 h, while the PPLO agar plates were incubated at 37 °C for 5–7 days in 5% CO2. Bacterial identification followed standard protocols, including colony morphology assessment; hemolysis type; Gram staining; and biochemical tests, such as oxidase, catalase, indole, urease, triple sugar iron (TSI), oxidation/fermentation, and motility tests [43].

Molecular identification of bacterial isolates and detection of specific virulence and resistance genes confirmed the presence of *Klebsiella pneumoniae* (16–23 S ITS) [44], *Klebsiella iutA* [28], *Klebsiella fimH* [45], *P. multocidaKmt1* [46], *Mannheimia haemolytica ssa* [47], *Pseudomonas 16 S rDNA* [48], *Pseudomonas toxA* [49], *Mycoplasma 16 S Rrna* [50], *Streptococcus 16 S rRNA* [51], *and E. coli phoA* [52].

### PCRbased bacterial detection

#### DNA extraction

DNA was extracted via the QIAamp DNA Mini Kit (Qiagen, Germany) with minor modifications according to the manufacturer’s protocol. Briefly, 200 µL of sample suspension was incubated with 10 µL of proteinase K and 200 µL of lysis buffer at 56 °C for 10 min. After adding 200 µL of ethanol, the samples were washed and centrifuged, and the nucleic acids were eluted with 100 µL of elution buffer.

The oligonucleotide primers used were supplied by Metabion (Germany) and are listed in Table 1.

**Table 1:** Primer sequences, target genes, amplicon sizes and cycling conditions for bacteria and some virulence genes

Target gene	Primers sequences	Amplified segment (bp)	Primary denaturation	Amplification (35 cycles)			Final extension	Reference
				Secondary denaturation	Annealing	Extension		
***Klebsiella. pneumoniae 16–23 S ITS***	ATTTGAAGAGGTTGCAAACGAT	130	94˚C 5 min	94˚C 30 s.	55˚C 30 s	0.72˚C 30 s.	72˚C 7 min	Turton et al. 2010 [44]
	TTCACTCTGAAGTTTTCTTGTGTTC							
***Klebsiella iutA***	GGCTGGACATGGGAACTGG	300	94˚C 5 min	94˚C 30 s.	63˚C 30 s.	72˚C 30 s.	72˚C 7 min	Yaguchi et al. 2007 [28]
	CGTCGGGAACGGGTAGAATCG							
***Klebsiella**** fimH*	TGCAGAACGGATAAGCCGTGG	508	94˚C 5 min.	94˚C 30 s.	50˚C 40 s	72˚C 45 s	72˚C 10 min	**Ghanbarpour and Salehi**,** 2010 ** [45]
	GCAGTCACCTGCCCTCCGGTA							
	GTAAATGCACTTGCTTCAGGAC							
***Pseudomonas 16 S rDNA***	GACGGGTGAGTAATGCCTA	618	94˚C 5 min.	94˚C 30 s.	50˚C 40 s.	72˚C 45 s	72˚C 10 min.	Spilker et al. 2004 [48]
	CACTGGTGTTCCTTCCTATA							
	CGCTGGCCCATTCGCTCCAGCGCT							
***E. coli phoA***	CGATTCTGGAAATGGCAAAAG	720	94˚C 5 min	94˚C 30 s.	55˚C 40 s	72˚C 45 s	. 72˚C 10 min	Hu et al. 2011 [52]
	CGTGATCAGCGGTGACTATGAC							
	ccggccatattcacataa							
***P. multocida*** ***Kmt1***	ATCCGCTATTTACCCAGTGG	460	94˚C 5 min.	94˚C 30 s.	55˚C 40 s	72˚C 45 s.	72˚C 10 min.	Duan et al. 2024 [46]
	GCTGTAAACGAACTCGCCAC							
***Mannheimia haemolytica ssa***	TTCACATCTTCATCCTC	325	94˚C 5 min.	94˚C 30 s.	50˚C 40 s.	72˚C 40 s.	72˚C 10 min	Hawari et al. 2008 [47]
	TTTTCATCCTCTTCGTC							
**Mycoplasma** ***16 S rRNA***	GGGAGCAAACAGGATTAGATACCCT	280	94˚C 5 min	94˚C 30 s.	55˚C 30 s.	72˚C 30 s.	72˚C 7 min.	Van Kuppeveld et al. 1994 [50]
	TGCACCATCTGTCACTCTGTTAACCTC							
**Streptococcus** ***16 S rRNA***	CGGGGGATAACTATTGGAAACGATA	912		94˚C 30 s.	55˚C 40 s.	72˚C 1 min.	72˚C 10 min.	Osakabe et al. 2006 [51]
	ACCTGTCACCCGATGTACCGAAGTA							

### PCR amplification

#### Uniplex PCR

The primers were utilized in a 25-µL reaction containing 12.5 µL of EmeraldAmp Max PCR Master Mix (Takara, Japan), 1 µL of each primer at a concentration of 20 pmol, 5.5 µL of water, and 5 µL of the DNA template. Amplifications were conducted in an Applied Biosystems 2720 thermal cycler.

#### Analysis of PCR products

The products of PCR were separated via electrophoresis on a 1.5% agarose gel (Applichem, Germany, GmbH) in 1x TBE buffer at room temperature via gradients of 5 V/cm. For gel analysis, 20 µL of the uniplex PCR products and 40 µL of the duplex PCR products were loaded into each gel slot. A GelPilot 100 bp plus ladder (Qiagen, gmbh, Germany) and a Generuler 100 bp ladder (Fermentas, Germany) were used to determine the fragment sizes. The gel was photographed with a gel documentation system (Alpha Innotech, Biometra), and the data were analysed with computer software.

Positive controls included *Klebsiella pneumoniae* ATCC^®^ BAA-1705D-5™, *Pasteurella multocida subsp. multocida* (ATCC^®^ 43137™), *Mannheimia haemolytica* (ATCC^®^ 33396™), *Pseudomonas aeruginosa* (ATCC^®^27853™), *Mycoplasma ovipneumoniae* (ATCC^®^ 29419™), *Streptococcus* sp. ATCC ^®^ 15,913, and *E. coli* ATCC 25,922. For virulence genes, positive and/or negative controls were field isolates supplied by the Reference Laboratory for Veterinary Quality Control on Poultry Production, Animal Health Research Institute.

### Nucleotide sequence variants between healthy and pneumonic ewes

#### DNA extraction and polymerase chain reaction (PCR)

A Gene JET whole blood genomic DNA extraction kit was used to extract genomic DNA from whole blood in accordance with the manufacturer’s instructions (Thermo Scientific, Lithuania). A Nanodrop was used to test high-quality, pure, and concentrated DNA. The immune (IL-1α, IL1B, IL6, TNF-α, IL10, LFA-1, CR2, IL17, IL13, DEFB123, SCART1, and ICAM1) and antioxidant (SOD1, CAT, GPX1, NOS, HMOX1, and NQO1) fragments were amplified via PCR. The primer sequences were created on the basis of the *Ovis aries* sequence published in PubMed. Table 2 lists the primers that were utilized in the amplification.

**Table 2:** Forward and reverse primer sequences, lengths of the PCR products and annealing temperatures of the investigated genes used for PCR-DNA sequencing

Gene	Forward	Reverse	Annealing temperature (°C)	Length of PCR product (bp)	Reference
*IL-1α*	5′-ATGGCCAAAGTCCCTGACCTC-3′	5′-ATGTAGCAGCCGTCATGTATGG-3′	60	472	Current study
*IL-1β*	5′-GAACCTTCATTGCCCAGGTTTC − 3′	5′-ACTGACTGCACGGCTGCATCA − 3′	58	388	Current study
*IL-6*	5′-GAGAGCTCCATCAGCCCTCCAG-3′	5′-GGAGTGGTTATTAGACCTGCGA-3′	62	526	Current study
*TNF-α*	5′-CAGACCAAGGTCAACATCCTCT-3′	5′-CAGGCCTCACTTCCCTACATC-3′	62	388	Current study
*IL-10*	5′-ACAACAGGGGCTTGCTCTTGC-3′	5′-CTCTCTTGGAGCATATTGAAGAC-3′	60	526	Current study
*LFA-1*	5′-TTCCTACACCATCGTGATGAG-3′	5′- ATACATTCCTGATAACCAGGGT-3′	60	438	Current study
*CR2*	5′-TAGCAGCTTAGGGACAGCTCT-3′	5′-CTATTGTAATATTCACAGATA-3′	58	381	Current study
*IL-17*	5′-ATGGCGTCTATGAGAACTGCCT-3′	5′-TTAAGCCACATGGCGGACAAT-3′	62	462	Current study
*IL-13*	5′-CTTAGGCCAGCCTATGCGTCTG-3′	5′- TGGTGTCTCGGACGTACTCACT-3′	58	376	Current study
*DEFB123*	5′- CAGAGCACTCCACCATTAGCT − 3′	5′- TTATTGATAGAGCTCAAGAATC − 3′	62	371	Current study
*SCART1*	5′-CAGAGCACTCCACCATTAGCT-3′	5′- GGCTCTGTCCCCACGCAGTGC-3′	62	220	Current study
*ICAM1*	5′-CGCCTCCGCCTGCGATGGCTC-3′	5′- CTTCACCCACAGGCTGCCAGAGT-3′	60	399	Current study
*SOD1*	5′-GCGTCGGCGTGTGTTCTGCGG-3′	5′-ATCTACGATATCCACAATGGCA-3′	62	387	Current study
*CAT*	5′-TCTGCAGCGCCGCTCAGACAC-3′	5′-TGGAGTATCTGGTAATGTCATG-3′	60	308	Current study
*GPX1*	5′-AGCTCACTGCTCTCAACTTGGA-3′	5′- ACGTGATGAACTTAGGGTCGGT − 3′	60	494	Current study
*NOS*	5′-GGCACGAGCGCCCGTCCGCCC-3′	5′-TCGCTCTCTCGGAGGTGGTAG-3′	58	540	Current study
*HMOX1*	5′-AGCACTGGTGACACCTTCTAG-3′	5′- TCGGCCACAATATCTGGGCTC-3′	62	387	Current study
*NQO1*	5′-CTACGCAGCCGCAGGATGGAGC-3′	5′- GCTCCATCCTGCGGCTGCGTAGAACAT-3′	64	377	Current study

*--IL-1α* Interleukin 1 alpha, *IL-1β* Interleukin 1 beta, *IL-6* Interleukin 6, *TNF- α* Tumor necrosis factor- alpha, *IL10* Interleukin 10, *LFA-1* Lymphocyte function-associated antigen 1, *CR2* Complement C3d receptor 2, *IL-17* Interleukin 17, *IL-13* Interleukin 13, *DEFB123* β-defensin, *SCART1* Scavenger Receptor Family Member Expressed On T Cells 1, *ICAM1* intercellular adhesion molecule 1, *SOD1* Superoxide dismutase 1, *CAT* Catalase, *GPX1* Glutathione peroxidase 1, *NOS* Nitric oxide synthetase, *HMOX1* Heme Oxygenase 1, *NQO1* NAD(P)H Quinone Dehydrogenase 1

In a thermal cycler, the polymerase chain reaction mixture was prepared at a final volume of 100 µL. Five microliters of DNA, 68 µL of d.d. water, 25 µL of PCR master mix (Jena Bioscience, Germany), and one µL of each primer were included in each reaction volume. For eight minutes, the reaction mixture was exposed to an initial denaturation temperature of 94 °C. As indicated in Table 2, the cycling process involved 30 cycles at 94 °C for 1 min for denaturation, 45 s for annealing, 45 s for extension at 72 °C, and a final extension at 72 °C for 8 min. The samples were stored at 4 °C, and agarose gel electrophoresis was used for identification. The samples were stored at 4 °C, and agarose gel electrophoresis was used to identify representative PCR analysis results. A gel documentation system was then used to visualize the fragment patterns under ultraviolet light.

#### DNA sequencing and polymorphism detection

The removal of primer dimers, nonspecific bands, and other contaminants was performed prior to DNA sequencing. Using a PCR purification kit and the manufacturer’s instructions, PCR products of the desired size (target bands) were purified as [53] described previously (Jena Bioscience # pp-201×s/Germany). The PCR product was quantified via a Nanodrop (UV‒Vis spectrophotometer Q5000/USA) to guarantee sufficient concentrations and purity of the PCR products and to produce high yields [54]. PCR products with the target band were sent for forward and reverse DNA sequencing via an ABI 3730XL DNA sequencer (Applied Biosystem, USA), which relies on the enzymatic chain terminator technique developed by [55], to detect SNPs in genes examined in both healthy controls and pneumonic ewes.

The software programs Chromas 1.45 and blast 2.0 were used to analyse the DNA sequencing data [56]. Single-nucleotide polymorphisms (SNPs) are defined as differences between the PCR products of the genes under study and between the PCR products and reference sequences found in GenBank. On the basis of DNA sequencing data alignment, the MEGA4 software package was used to compare the amino acid sequences of the genes under investigation among the enrolled ewes [57].

#### Total RNA extraction, reverse transcription and quantitative real-time PCR

TRIzol reagent was used to extract total RNA from ewe blood in accordance with the manufacturer’s instructions (RNeasy Mini Ki, Catalogue no. 74104). A NanoDrop^®^ ND-1000 spectrophotometer was used to quantify and quantify the amount of isolated RNA. Each sample’s cDNA was produced in accordance with the manufacturing technique (Thermo Fisher, Catalogue no. EP0441). Using quantitative RT‒PCR with SYBR Green PCR Master Mix (2x SensiFastTM SYBR, Bioline, CAT No: Bio98002), the gene expression patterns of genes encoding immunity and antioxidants were evaluated. Using SYBR Green PCR Master Mix (Quantitect SYBR Green PCR Kit, Catalogue no. 204141), real-time PCR was used to measure the relative amount of mRNA. As indicated in Table 3, primer sequences were created using the Ovis aries sequence published in PubMed.

**Table 3:** Oligonucleotide primer sequences, accession numbers, annealing temperatures and PCR product sizes of the investigated genes via real-time PCR

Gene	Primer	Product length (bp)	Annealing Temperature (°C)	Accession number	Source
*IL-1α*	F5′- CCATACATGACGGCTGCTACA − 3 R5′- TGTCTCAGGCATCTCCTTATGC − 3′	180	62	NM_001009808.1	Current study
*IL-1β*	F5′- ACAGATGAAGAGCTGCACCC − 3′ R5′- AGACATGTTCGTAGGCACGG − 3′	161	58	NM_001009465.2	Current study
*IL-6*	F5- TGCAGTCCTCAAACGAGTGG − 3′ R5′- CCGCAGCTACTTCATCCGAA − 3′	110	62	NM_001009392.1	Current study
*TNF-α*	F5′- CTTTGGGATCATCGCCCTGT − 3′ R5′- CAGCCCTGAGCCCCTAATTC − 3′	129	60	NM_001024860.1	Current study
*IL-10*	F5′- CTCGACTAGACTCCCCGACA − 3′ R5′- ACTCTAGGGGAGAGGCACAG − 3′	102	58	NM_001009327.1	Current study
*LFA-1*	F5′- TTGGGTACCGTGTTCTGCAA − 3′ R5′- TCACATGTTCGAGACAGCCC − 3′	223	58	NM_001035124.1	Current study
*CR2*	F5′- TACCCTAGAAGGCAGTCCCC-3′ R5′- AAGGTGACAACACCCAAGCA G − 3′	153	60	NM_001009724.1	Current study
*IL-17*	F5′- TTATCACAAGCGCTCCACCT − 3′ R5′- GTGATGGTCCACCTTCCCTT − 3′	133	58	XM_004018887.5	Current study
*IL-13*	F5′- TCCAGCACTAAAGCAGTGGG − 3′ R5′- TGCTGGCTGTCAGACAAGAG − 3′	141	62	NM_001082594.1	Current study
*DEFB123*	F5′- ACTGTGCTGTGTGAAGTCCA − 3′ R5′- GGGGTTAGACTCAGGGTAGC − 3′	112	58	XM_027976443.1	Current study
*SCART1*	F5′- CCACTGGGACTTGGCAGAC − 3′ R5′- GCTCTGTCCCCACGCA − 3′	140	58	EF215856.1	Current study
*ICAM1*	F5′- CACCATATACTGGTTCCCGGAG − 3 R5′- CATGGTCCTCTCTTCTCGGC − 3′	224	62	NM_001009731.1	Current study
*SOD1*	F5- TGATCATGGGTTCCACGTCC − 3′ R5′- CACATTGCCCAGGTCTCCAA − 3′	139	60	NM_001145185.2	Current study
*CAT*	F5- TTCGCTTCTCCACTGTTGCT − 3′ R5′- CCGGATCCTTCAGGTGTGTC − 3′	207	58	XM_004016396.5	Current study
*GPX1*	F5- AACGTAGCATCGCTCTGAGG − 3 R5- CAAACTGGTTGCACGGGAAG − 3	115	62	XM_004018462.5	Current study
*NOS*	F5′- CTCCCAAGGTGACTTCCCAGA − 3′ R5′- ACCAAAGGGCTGACTGTAGG − 3′	142	58	NM_001129901.1	Current study
*HMOX1*	F5′- CAAGCGCTATGTTCAGCGAC − 3′ R5′- GCTTGAACTTGGTGGCACTG − 3′	206	60	MK630326.1	Current study
*NQO1*	F5′- GGCAGTGGCTCCATGTACTC − 3′ R5′- TAGATGGCCACCTCACATGC − 3′	116	62	XM_012190046.4	Current study
*ß. actin*	F5′- GCAAAGACCTCTACGCCAAC-3′ R5′- TGATCTTCATCGTGCTGGGT − 3′	114	60	NM_001009784.3	Current study

*-IL-1α* Interleukin 1 alpha, *IL-1β* Interleukin 1 beta, *IL-6* Interleukin 6, *TNF- α* Tumor necrosis factor- alpha, *IL10* Interleukin 10, *LFA-1* Lymphocyte function-associated antigen 1, *CR2* Complement C3d receptor 2, *IL-17* Interleukin 17, *IL-13* Interleukin 13, *DEFB123* β-defensin, *SCART1* Scavenger Receptor Family Member Expressed On T Cells 1, *ICAM1* intercellular adhesion molecule 1, *SOD1* Superoxide dismutase 1, *CAT* Catalase, *GPX1* Glutathione peroxidase 1, *NOS* Nitric oxide synthetase, *HMOX1* Heme Oxygenase 1, *NQO1* NAD(P)H Quinone Dehydrogenase 1

A constitutive control for normalization was the housekeeping gene ß-actin. Three microliters of total RNA, four microliters of 5x Trans Amp buffer, 0.25 µL of reverse transcriptase, 0.5 µL of each primer, 12.5 µL of 2x Quantitect SYBR green PCR master mix, and 8.25 µL of RNase-free water made up the reaction mixture, which had a total volume of 25 µL. Reverse transcription was performed at 50 °C for 30 min, initial denaturation at 94 °C for 10 min, 40 cycles of 94 °C for 15 s, the annealing temperatures indicated in Table 3, and 72 °C for 30 s. A melting curve analysis was carried out at the conclusion of the amplification phase to verify the specificity of the PCR products. The 2^−ΔΔCt^ technique was used to calculate the relative expression of each gene per sample in comparison to that of the *ß.actin* gene [58].

### Immunological and antioxidant parameters

In accordance with the author’s guidelines, the obtained plasma and serum samples were used to evaluate the following parameters: ELISA kits from MyBiosecure Company^®^ were used for the evaluation of serum pro-infammatory cytokines (IL-1α, IL-1β, IL-6, TNF-α) and anti-inflammatory cytokines (IL-10), serum concentrations of free radicals (nitric oxide (NO), malondialdehyde (MDA)), antioxidants (catalase (CAT), glutathione peroxidase (GPx), glutathione reductase (GR)), (spectrophotometrically using commercial kits from Biodiagnostic Company^®^), serum complement-3 (C3) and complement-4 (C4) (ELISA technique using commercial kits from New Test Company^®^), and immunoglobulin (Igs) serum concentrations (IgG, IgM, IgA) (Turbidmetrically using kits supplied by Biorex Diagnostics^®^, UK).

### Statistical analysis

Independent-samples t tests were used to compare the means of the measured variables between the PG and HCG via SPSS version 23. The difference was considered significant at *P* < 0.05. All variables of the PG and HCG are presented as the means ± SDs. Pearson’s simple correlation test was used to determine correlations between the genetic, immunological and antioxidant parameters. The cut-off points, sensitivity, specificity and likelihood ratios (LRs) for the measured cytokines were estimated in the PG and HCG via the graphed prism version 8 program. The positive predictive value (PPV), negative predictive value (NPV), accuracy rate and percentages of increase were calculated via the following equations:

$$\mathrm{PPV}=\mathrm{True}\;\mathrm{positive}\div\mathrm{Total}\;\mathrm{positive}\;\ast\;100$$

$$\mathrm{NPV}=\mathrm{True}\;\mathrm{negative}\div\mathrm{Total}\;\mathrm{negative}\;\ast\;100.$$

$$\begin{aligned} \mathrm{Accuracy}\;\mathrm{rate}=\ & (\mathrm{True}\;\mathrm{positive}+\mathrm{True}\;\mathrm{negative})\\&\div\mathrm{Total}\;\mathrm{population}\;\ast\;100 \end{aligned}$$

$$\begin{aligned} \mathrm{Percentage}\;&\mathrm{of}\;\mathrm{increaese}\\&=(\mathrm{The}\;\mathrm{mean}\;\mathrm{value}\;\mathrm{of}\;\mathrm{the}\;\mathrm{marker}\;\mathrm{concentration}\;\mathrm{in}\;\;\mathrm{DG}\;\\&-\mathrm{The}\;\mathrm{mean}\;\mathrm{value}\;\mathrm{of}\;\mathrm{its}\;\mathrm{concentration}\;\mathrm{in}\;\mathrm{CG})\\&\div\mathrm{The}\;\mathrm{mean}\;\mathrm{value}\;\mathrm{of}\;\mathrm{its}\;\mathrm{concentration}\;\mathrm{in}\;\mathrm{CG}\;\times100 \end{aligned}$$

## Results

### Clinical findings

This study demonstrated that infected sheep typically display signs of respiratory distress, including rhinitis, clogged mucous membranes, and a wet, harsh cough. The most common symptoms were elevated rectal temperature (over 41 °C), serous or mucoid nasal discharge, and increased respiratory and pulse rates. Additionally, ataxia, depression, ruminal atony, and abnormal milk production were observed. Early-stage auscultation revealed pleuritic friction rubs, which later progressed to muffled sounds in the more severe stages.

### Isolation and identification

Bacterial isolates were detected in 80 out of 150 cases (53.3%). All bacterial isolates were subjected to PCR assays to identify specific genes via targeted primers, with positive control DNA samples incorporated into each reaction.

#### Prevalence of bacterial species isolated from infected animals

Table 4 summarizes the bacterial species identified from pneumonic cases in sheep via traditional bacteriological methods. The species isolated from sheep, nasal swabs and lung samples included *Klebsiella pneumoniae* (35% & 50%), *Pasteurella multocida* (28% & 30%), *Mannheimia haemolytica* (32% & 38%), *Pseudomonas* spp. (12% & 15%), *Mycoplasma* (30% & 72%), *Streptococcus* (25% & 38%), and *Escherichia coli* (12% & 36%).

**Table 4:** Isolation of bacterial species from pneumonic sheep

Bacterial infection	No. of isolated bacteria		Percentage %		Total 150	Percentage %
	Nasal swab 100	Lung 50	Nasal swab	Lung		
*Klebsiella pneumoniae*	35	25	35	50	60	40
*Pasteurella. multocida*	28	15	28	30	43	28.6
*Mannheimia haemolytica*,	32	19	32	38	51	34
*Pseudomonas aeruginosa*	12	15	12	30	27	18
*Mycoplasma*	30	36	30	72	66	44
*Streptococcus pneumoniae*,	25	19	25	38	44	29,3
*Escherichia coli*	12	18	12	36	30	20

### Molecular identification and detection of several virulence genes

**Fig. 1:**
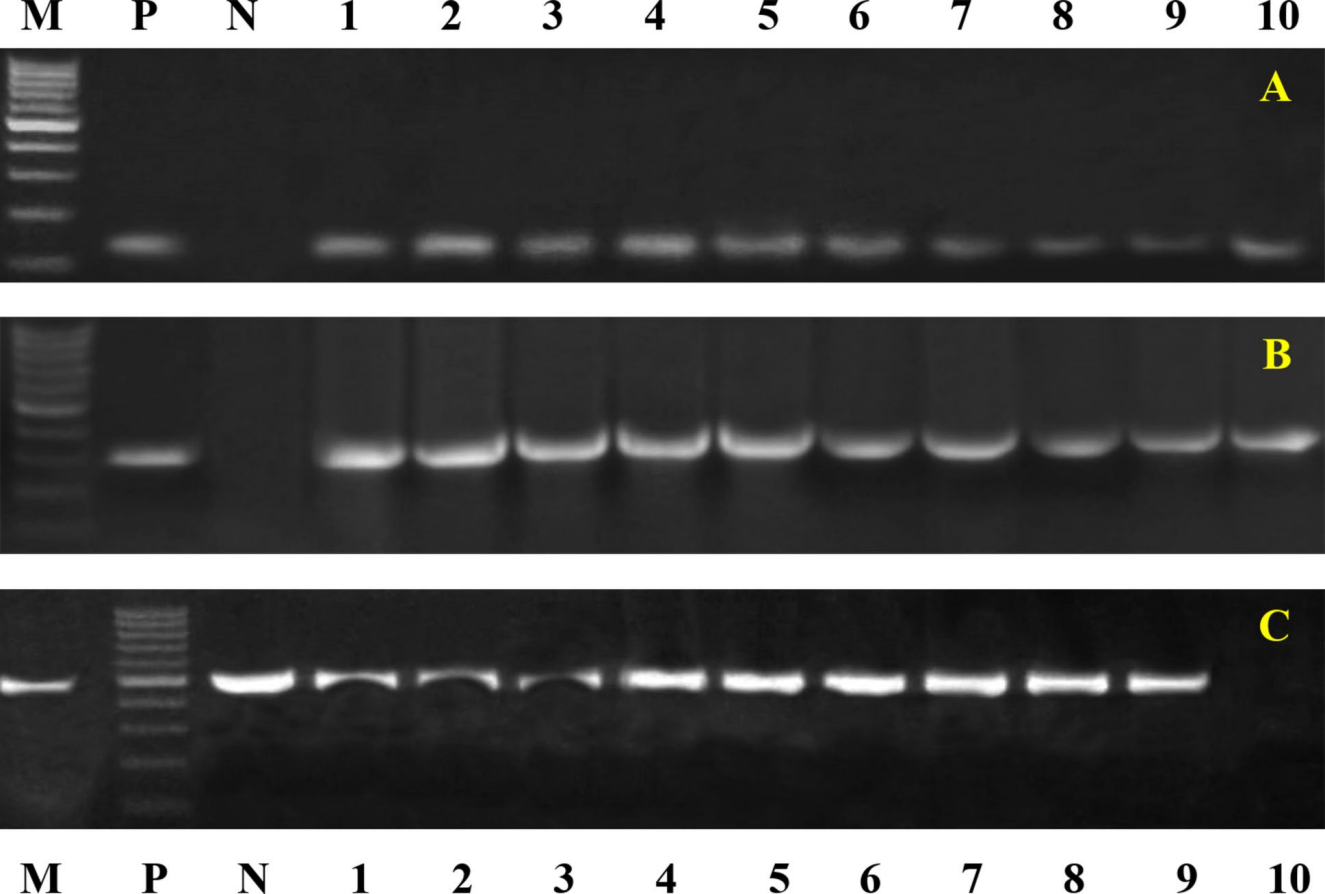
Representative gel electrophoresis of *K. pneumoniae* strains isolated from sheep with pneumonia. **A** *16S–23S ITS* gene (genetic marker *of K. pneumoniae*), with an expected band size of 130 bp. **B** *iutA* gene with a band size of 300 bp. This gene was represented in 39% of the isolated *K. pneumoniae* strains. **C** *fimH* gene with the expected band size of 508 bp. This gene was present in 68% of the isolates

**Fig. 2:**
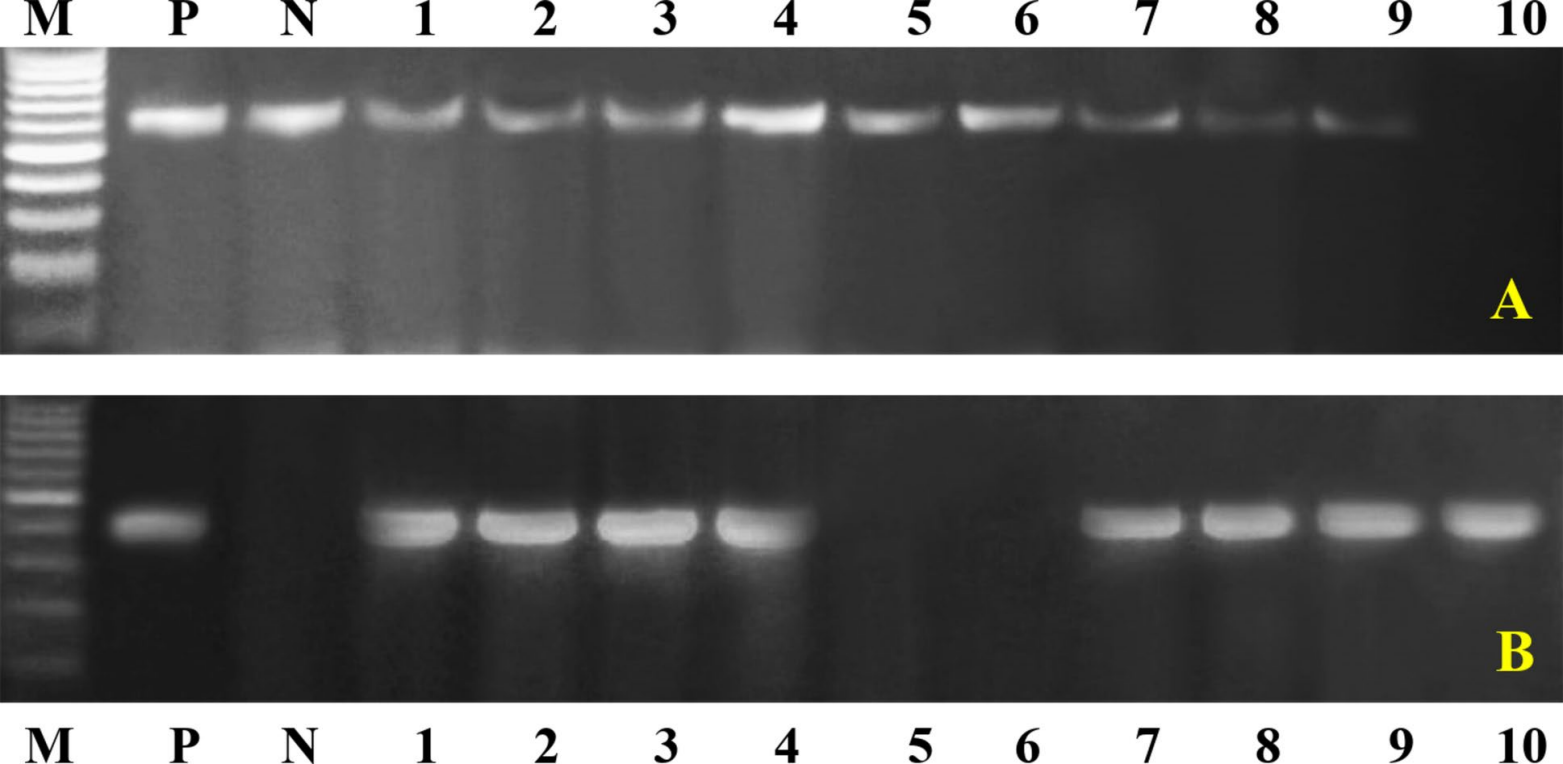
Representative gel electrophoresis of several virulence genes from Pseudomonas strains isolated from sheep with pneumonia **A** *16 S rDNA gene* (genetic marker Pseudomonas), with an expected band size of 618 bp. **B** *ToxA gene* with a band size of 396 bp. This gene was represented in 59.2% of the isolated Pseudomonas strains

**Fig. 3:**
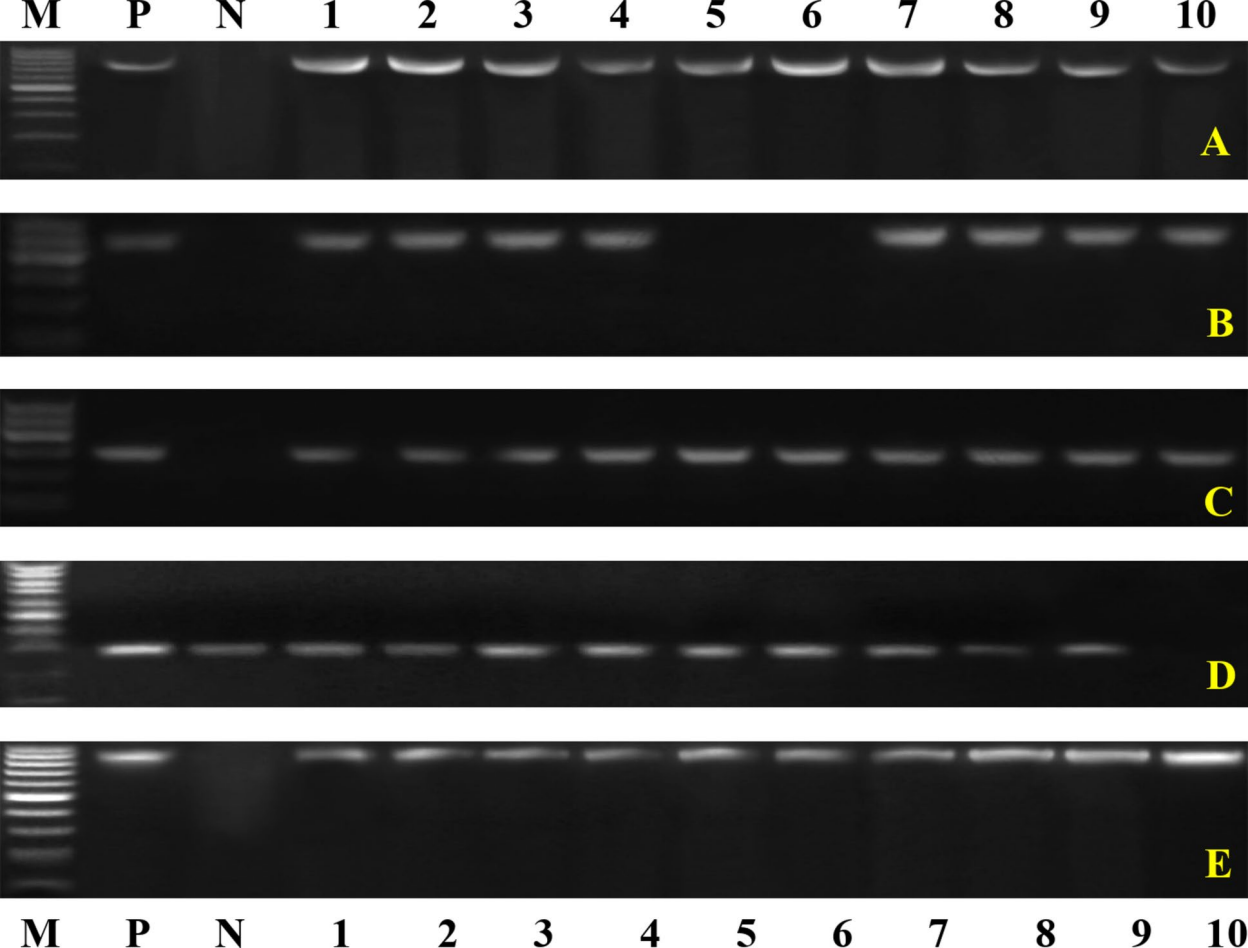
Disagnostic PCR for some bacterial diseases causing pneumonia in sheep. Agarose gel electrophoresis of the group-specific primer set of the **A** *phoA gene* (genetic marker *of E. coli*), with the expected band size of 720 bp. **B** *P. multocida Kmt1* target gene at 460 bp. **C** Group-specific primer set of *Mannheimia haemolytica ssa* at the expected band size of 508 bp. **D** Group-specific primer set for *Mycoplasma16S rRNA*, *with* the target gene at 280 bp. **E** The group-specific primer set for Streptococcus16S rRNA, with positive samples except the 912 bp Lane (M): DNA ladder. Lane (N): negative control. Lane (P): control positive Lanes (1--10) samples

Conventional PCR tests were conducted on biochemically and serologically identified bacterial isolates to confirm their identity and detect specific virulence genes. The results revealed the following: *Klebsiella pneumoniae* 16–23 S ITS (40%, Fig. 1A), the *Klebsiella* virulence gene iutA (39%, Fig. 1B), the fimH gene (68%, Fig. 1C), *Pseudomonas* 16 S rRNA (Fig. 2A), toxA (59.2%, Fig. 2B), *E. coli* phoA (Fig. 3A), *P. multocida Kmt1* (Fig. 3B), *Mannheimia haemolytica ssa* (Fig. 3C), *Mycoplasma 16 S rRNA* (Fig. 3D), and *Streptococcus* (Fig. 3E).

#### PCR-DNA sequencing of immunity-, antioxidant- and GIT health-related genes

PCR-DNA sequencing of the following genes revealed variations in their nucleotide sequences: IL-1α (472 bp), IL1B (388 bp), IL6 (526 bp), TNF-α (388 bp), IL10 (526 bp), LFA-1 (438 bp), CR2 (381 bp), IL17 (462 bp), IL13 (376 bp), DEFB123 (371 bp), SCART1 (220 bp), ICAM1 (399 bp), SOD1 (387 bp), CAT (308 bp), GPX1 (494 bp), NOS (540 bp), HMOX1 (387 bp), and NQO1 (377 bp) between healthy controls and pneumonic ewes. A comparison of pneumonic sheep with healthy controls revealed changes in exonic regions, resulting in altered DNA sequences. A total of 48 SNPs were identified, with 17 synonymous and 31 nonsynonymous mutations (Table 5).

**Table 5:** Distribution of SNPs and types of mutations in immune and antioxidant genes in healthy and pneumonia-affected sheep

Gene	SNPs	Healthy	Pneumonia	Total	Type of mutation	Amino acid number and type
*IL-1α*	T111C	21	-	21/100	Synonymous	37 D
	A122G	32	-	32/100	Non-synonymous	41 E to G
	T285C	-	26	26/100	Synonymous	95 D
*IL-1β*	C56T	-	17	17/100	Non-synonymous	19 S to L
*IL-6*	C66T	-	34	34/100	Synonymous	22 H
*TNF-α*	C174T	22	-	22/100	Synonymous	58 A
	C278T	-	28	28/100	Non-synonymous	93 S to L
	T330C	-	41	41/100	Synonymous	110 S
*IL-10*	G87C	38	-	38/100	Synonymous	29 P
	C137G	-	25	25/100	Non-synonymous	46 P to R
	G174A	-	36	36/100	Synonymous	58 Q
	T374C	33	-	33/100	Non-synonymous	125 M to T
	G404A	-	29	29/100	Non-synonymous	135 R to H
*LFA-1*	C85T	-	14	14/100	Non-synonymous	29 R to C
	A142G	43	-	43/100	Non-synonymous	48 S to G
	G154A	35	-	35/100	Non-synonymous	52 G to S
	T281C	-	24	24/100	Non-synonymous	94 L to P
*CR2*	C100G	37	-	37/100	Non-synonymous	34 L to V
	T111C	28	-	28/100	Synonymous	37 D
	T269A	34	-	34/100	Non-synonymous	90 L to M
*IL-17*	G72A	29	-	29/100	Synonymous	24 G
	T327C	-	31	31/100	Synonymous	109 N
	T360C	39	-	39/100	Synonymous	120 L
*IL-13*	C294A	-	36	36/100	Non-synonymous	98 H to Q
*DEFB123*	A56G	-	40	40/100	Non-synonymous	19 H to R
	T186G	-	21	21/100	Non-synonymous	62 H to Q
*SCART1*	C77G	42	-	42/100	Non-synonymous	26 P to R
*ICAM1*	C114T	-	31	31/100	Synonymous	38 L
	C232T	17	-	17/100	Non-synonymous	78 P to S
*SOD1*	T51C	36	-	36/100	Synonymous	17 S
	C196T	-	16	16/100	Synonymous	66 L
	G208T	24	-	24/100	Non-synonymous	70 D to Y
*CAT*	T64C	-	17	17/100	Synonymous	22 L
	C158T	-	44	44/100	Non-synonymous	53 A to V
*GPX1*	C62G	-	32	32/100	Non-synonymous	21 P to R
	C193A	13	-	13/100	Non-synonymous	65 H to N
	C256G	45	-	45/100	Non-synonymous	89 R to G
	C298T	33	-	33/100	Non-synonymous	100 R to C
	G391C	-	25	25/100	Non-synonymous	131 E to Q
*NOS*	G175A	19	-	19/100	Non-synonymous	59 A to T
	A244C	42	-	42/100	Non-synonymous	82 S to R
	C367T	30	-	30/100	Non-synonymous	123 P to S
	T457C	-	15	15/100	Non-synonymous	153 W to R
*HMOX1*	C33T	-	27	27/100	Synonymous	11 P
	C108T	44	-	44/100	Synonymous	36 A
	C352T	-	23	23/100	Non-synonymous	118 R to C
*NQO1*	G56T	25	-	25/100	Non-synonymous	19 R to L
	G244A	17	-	17/100	Non-synonymous	82 G to R

-A= Alanine; C= Cisteine; D= Aspartic acid; E= Glutamic acid; G= Glycine; H= Histidine; L= Leucine; M= Methionine; N= Asparagine; P= Proline; Q= Glutamine; R= Argnine; S= Serine; T= Threonine; V= Valine; W= Tryptophan; and Y= Tyrosine

*-IL-1α* Interleukin 1 alpha, *IL-1β* Interleukin 1 beta, *IL-6* Interleukin 6, *TNF- α* Tumor necrosis factor- alpha, *IL10* Interleukin 10, *LFA-1* Lymphocyte function-associated antigen 1, *CR2* Complement C3d receptor 2, *IL-17* Interleukin 17, *IL-13* Interleukin 13, *DEFB123* β-defensin, *SCART1* Scavenger Receptor Family Member Expressed On T Cells 1, *ICAM1* intercellular adhesion molecule 1, *SOD1* Superoxide dismutase 1, *CAT* Catalase, *GPX1* Glutathione peroxidase 1, *NOS* Nitric oxide synthetase, *HMOX1* Heme Oxygenase 1, *NQO1* NAD(P)H Quinone Dehydrogenase 1

#### Gene expression patterns of immune and antioxidant markers

**Fig. 4:**
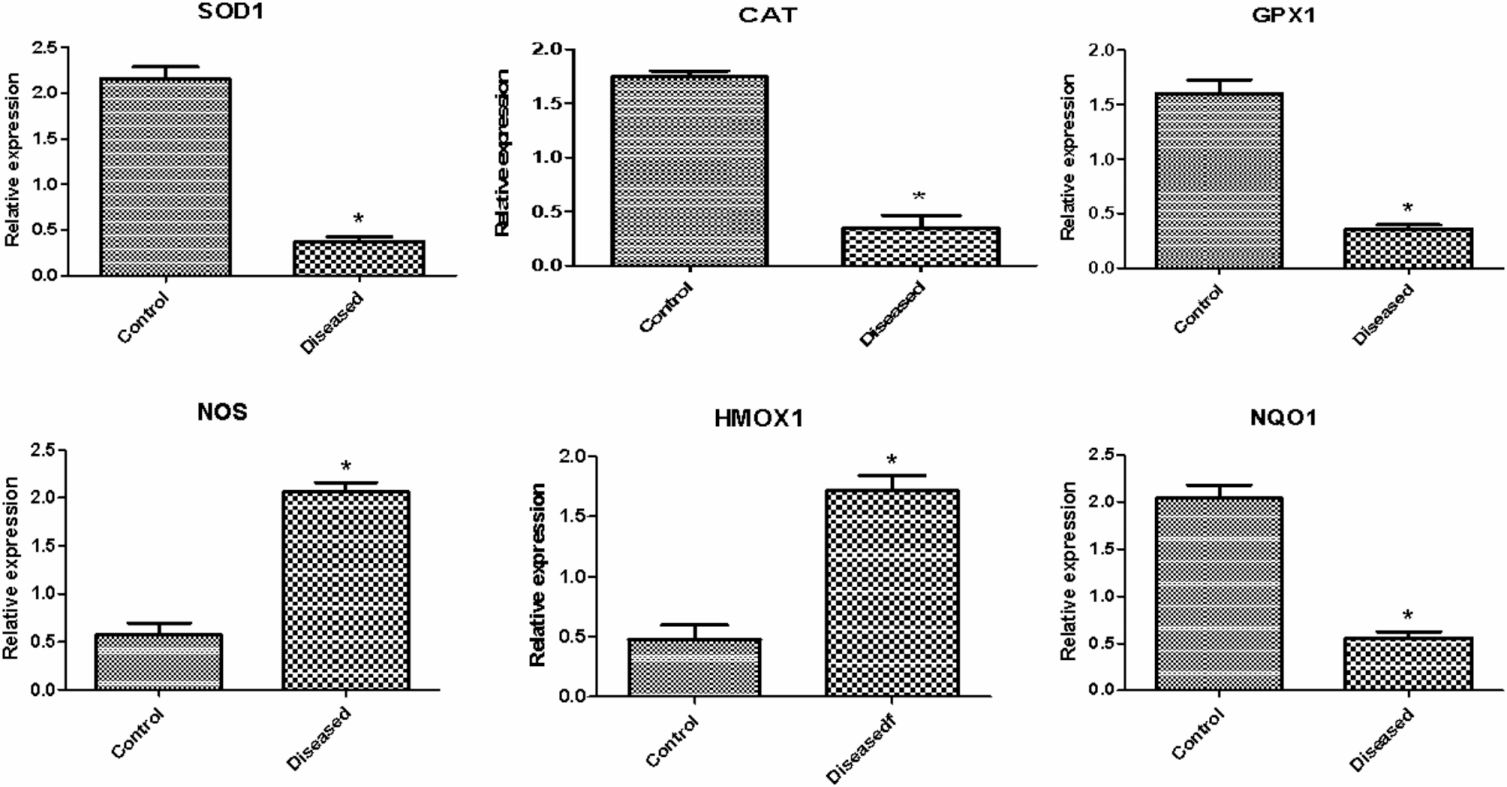
Antioxidant gene transcript levels in healthy and pneumonic sheep. The symbol * denotes significance when *p* < 0.05

**Fig. 5:**
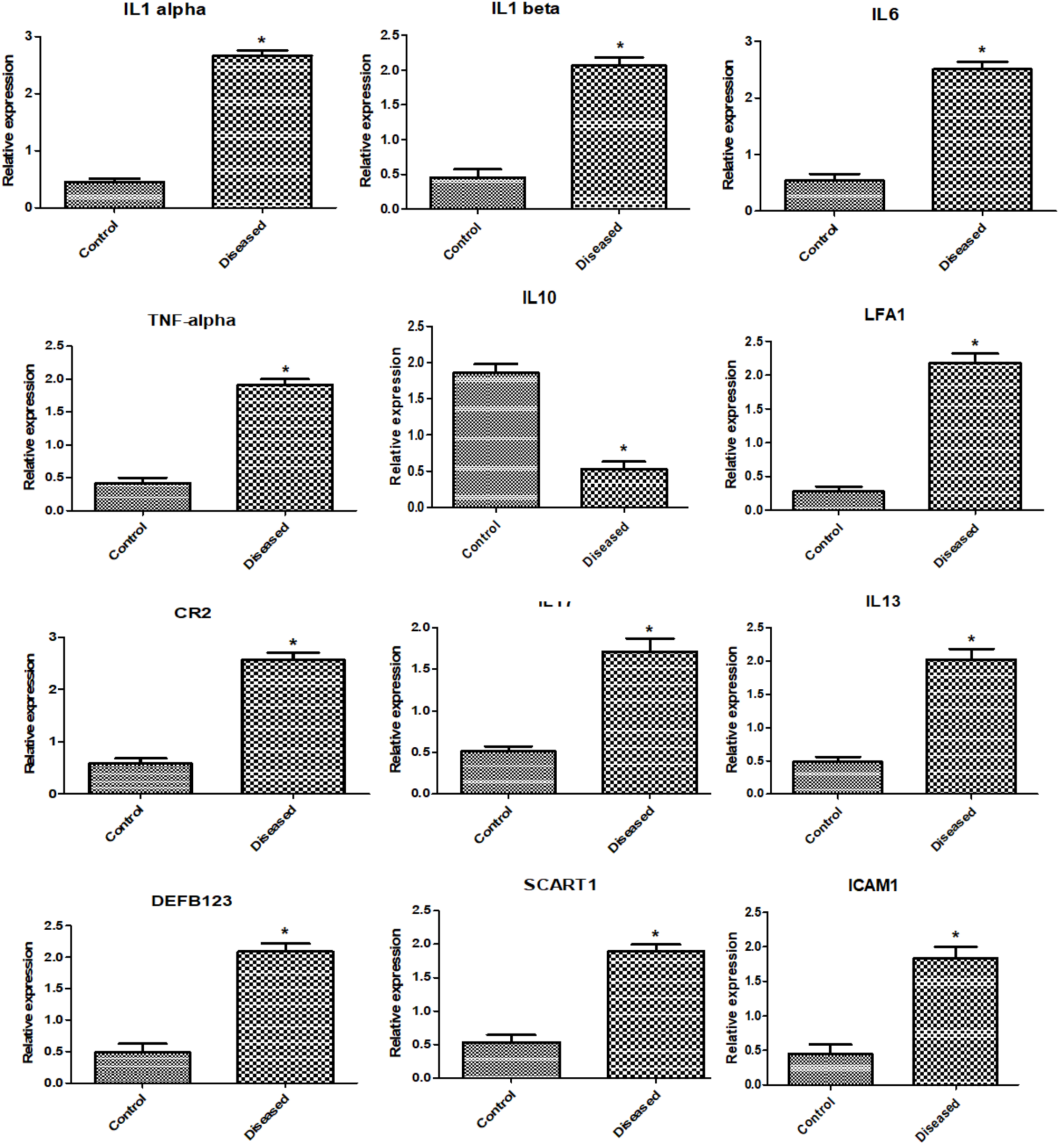
Immune gene transcript levels in healthy and pneumonic sheep. The symbol * denotes significance when *p* < 0.05

Figures 4 and 5 show the gene expression profiles of immunological and antioxidant markers. In sheep with pneumonia, the expression levels of *IL-1α*,* IL-1B*,* IL6*,* TNF-α*,* LFA-1*,* CR2*,* IL17*,* IL13*,* DEFB123*,* SCART1*,* ICAM1*,* NOS*, and *HMOX1* were significantly greater than those in healthy control sheep. Conversely, the expression levels of IL10, SOD1, CAT, GPX1, and NQO1 were significantly lower in the pneumonia group than in the healthy control group. The gene expression of immune and antioxidant markers was significantly related to pneumonia resistance/susceptibility. In healthy Barki ewes, the most downregulated gene was LFA-1 (0.27 ± 0.07), whereas the most upregulated gene was SOD1 (2.15 ± 0.13). In sheep with pneumonia, CAT (0.35 ± 0.11) was the most downregulated, whereas IL-1α (2.66 ± 0.08) was the most upregulated.

### Immunological and antioxidant profiles

Table 6 highlights the significant innate immune response in ewes with pneumonia, as evidenced by elevated levels of proinflammatory cytokines (IL-1α, IL-1β, IL-6, and TNF-α) and free radicals (NO and MDA) in the pneumonia group (PG) compared with those in the healthy control group (HCG). In contrast, compared with the HCG, the PG presented significantly lower levels of C3 and C4, antioxidants (CAT, GPx, and GR), and anti-inflammatory cytokines (IL-10). Notably, the PG resulted in significant hyperimmunoglobulinemia, indicating a robust humoral immune response. The cytokines and oxidative stress markers demonstrated high sensitivity, specificity, PPV, NPV, and accuracy rates (AUC = 1), except for NO, which showed low specificity and LR. The levels of proinflammatory cytokines, particularly IL-1α, were greater than those of oxidative stress markers (Table 7).

**Table 6:** Comparison of the immunological and antioxidant profile parameters between the pneumonic group and the control group. The values are the means ± SDs

Parameters	CG	PG
IL-1α (Pg/ml)	24.05 ± 3.34	95.81 ± 1.68^*^
IL-1β (Pg/ml)	25.99 ± 2.96	99.11 ± 0.65^*^
IL-6 (Pg/ml)	24.63 ± 2.92	86.36 ± 0.09^*^
TNF-α (Pg/ml)	24.91 ± 2.98	87.44 ± 1.60^*^
IL-10 (Pg/ml)	103.70 ± 3.31	90.69 ± 2.24^*^
C3 (mg/dl)	151.17 ± 5.29	110.49 ± 1.83^*^
C4 (mg/dl)	12.07 ± 0.60	7.14 ± 0.83^*^
IgG (mg/dl)	229.02 ± 19.74	350.26 ± 36.07^*^
IgM (mg/dl)	13.79 ± 1.43	39.26 ± 3.04^*^
IgA (mg/dl)	4.49 ± 0.83	8.43 ± 0.63^*^
MDA (nmol/ml)	12.95 ± 1.14	23.70 ± 1.51^*^
NO (µmol/L)	26.80 ± 1.20	32.33 ± 1.66^*^
CAT (U/L)	412.25 ± 14.64	289.20 ± 6.16^*^
GPx (mU/L)	1015.45 ± 2.95	742.87 ± 16.76^*^
GR (ng/ml)	8.15 ± 0.62	5.16 ± 0.44^*^

*IL-1α* Interleukin 1 alpha, *IL-1β* Interleukin 1 beta, *IL-6* Interleukin 6, *TNF- α* Tumor necrosis factor- alpha, *IL10* Interleukin 10, *C3* Complement 3, *C4* Complement 4, *IgG* Immunoglobulin G, *IgM* Immunoglobulin M, *IgA* Immunoglobulin A, *MDA* Malondialdehyde, *NO* Nitric oxide, *CAT* Catalase, *GPx* Glutathione peroxidase, *GR* Glutathione reductase

Significant differences between the two groups, were indicated by (*) when *P* < 0.05

**Table 7:** Cut-off points, sensitivity, specificity, LR, PPV, NPV, accuracy rate and percentages of increase or decrease in the estimated cytokines and oxidative stress markers in the PG compared with those in the CG

Parameters	Cut-off	Sensitivity	Specificity	PPV	NPV	LR	AR	% of increase or decrease
IL-1α (Pg/ml)	29.50	100%	90%	93.75%	100%	10	96%	298.38%
IL-1β (Pg/ml)	28.50	100%	85%	90.91%	100%	6.6	94%	281.34%
IL-6 (Pg/ml)	28.50	100%	90%	93.75%	100%	10	96%	250.63%
TNF-α (Pg/ml)	28.50	100%	90%	93.75%	100%	10	96%	251.02%
IL-10 (Pg/ml)	100.80	100%	80%	88.24%	100%	5	92%	−12.57%
MDA (nmol/ml)	14.10	100%	80%	88.24%	100%	5	92%	83.01%
NO (µmol/L)	27.50	100%	60%	78.95%	100%	2.5	84%	20.63%
CAT (U/L)	392.50	100%	90%	93.75%	100%	10	96%	−29.85%
GPx (mU/L)	1012.00	100%	90%	93.75%	100%	10	96%	−26.81%
GR (ng/ml)	7.59	100%	80%	88.24%	100%	5	92%	−36.69%

LR = 0.5–5: low; LR = 5–10: moderate; LR > 10: high

### Pathological findings

**Fig. 6:**
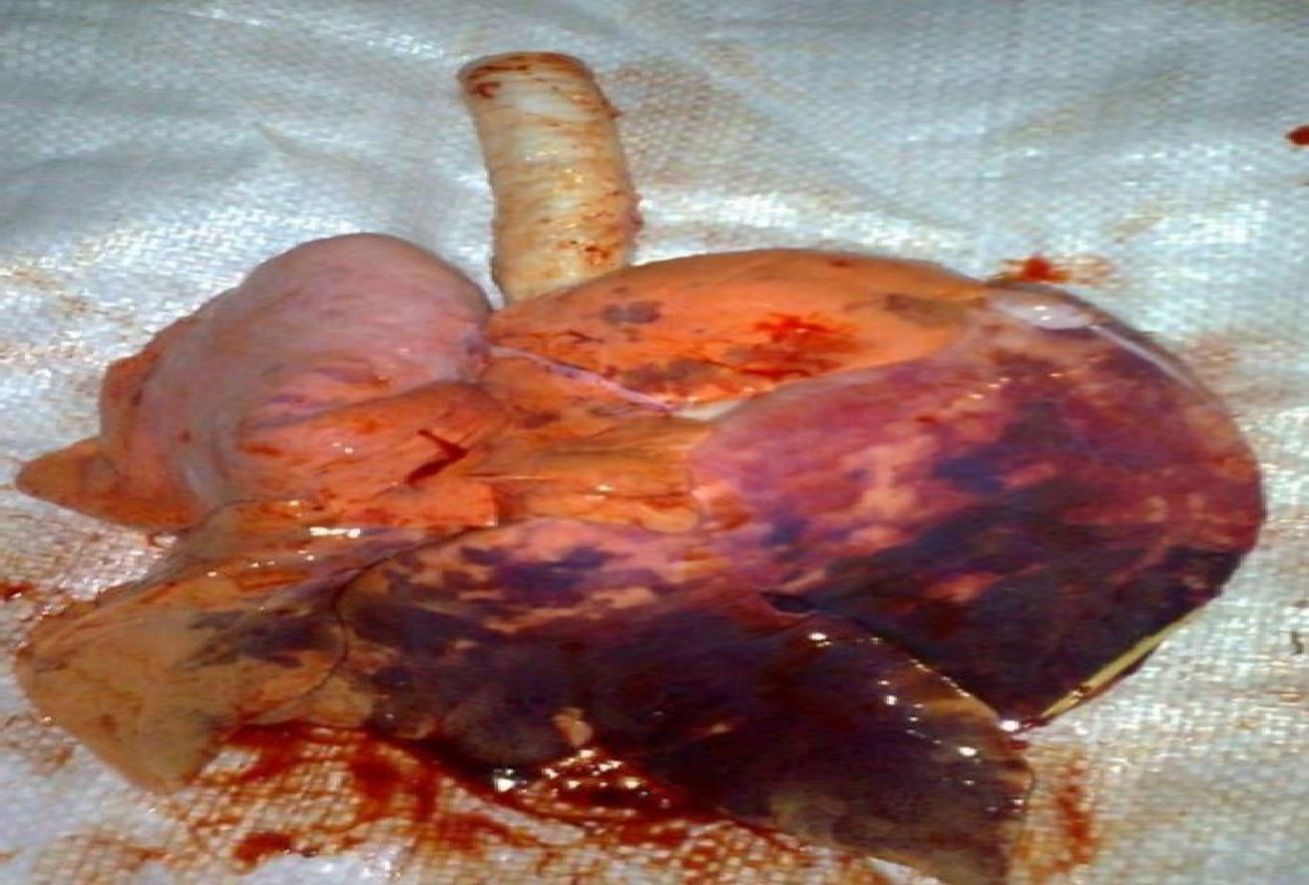
Languages infected with congestion, hepatization, and marbling appearance

**Fig. 7:**
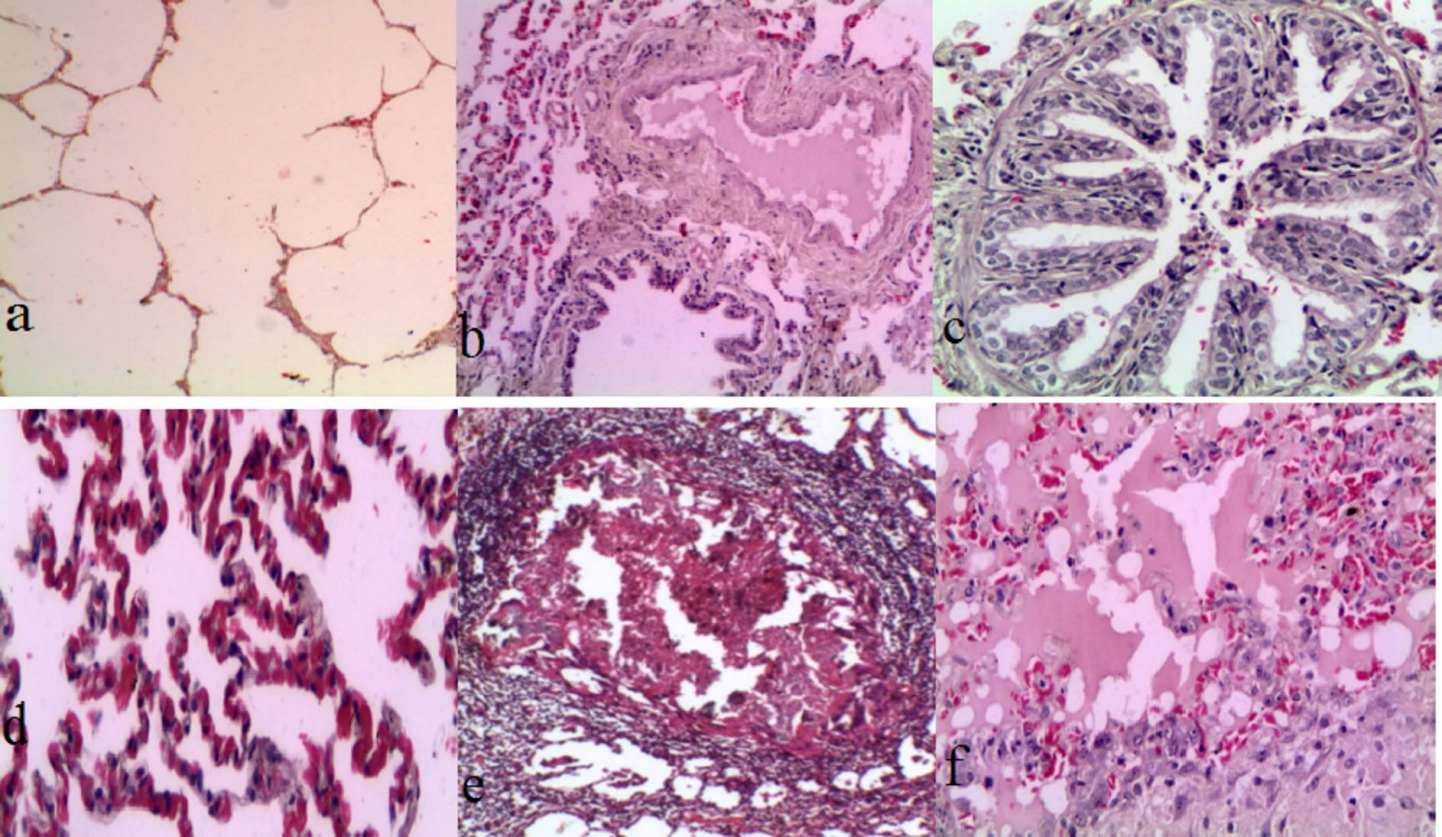
Lung sheep. -positive samples in the pneumonia stage: **a** Histopathological examination of the lung showing alveolar emphysema and giant alveoli and **b** histopathological examination of the lung showing cattarhal bronchiolitis, edema and congestive atelectasis. **c** Histopathological examination of the lung showing cataract hyperplasia resulting from epithelial linning and the elimination of epithelial cells inside the lumen. **d** Histopathological examination of the lung showing active alveolar hyperemia and atelectasis. **e** Histopathological examination of a lung showing a granuloma. **f** Histopathological examination of a lung showing serous pneumonia and congestion (H&E× 4)

The affected lungs exhibited pronounced irregular consolidation with a lobular or lobar to diffuse pattern, primarily in the cranioventral to caudal lobes. The areas of consolidation varied in color from dark red to gray, pink, and marbled hues (Fig. 6). Histopathological examination revealed alveolar emphysema and giant alveoli in 47 out of 50 (94%) lung samples (Fig. 7a); catarrhal bronchiolitis, edema, and congestive atelectasis in 30 lung samples (60%) (Fig. 7b); catarrhal bronchitis, hyperplasia of the epithelial lining, and desquamated epithelial cells in 35 lung samples (70%) (Fig. 7c); active alveolar hyperemia and atelectasis in 35 lung samples (80%) (Fig. 7d); granuloma formation in 48 lung samples (96%) (Fig. 7e); and serous pneumonia with congestion in 38 lung samples (76%) (Fig. 7f).

### Correlations between gene expression patterns and immune and antioxidant marker serum profiles

**Fig. 8:**
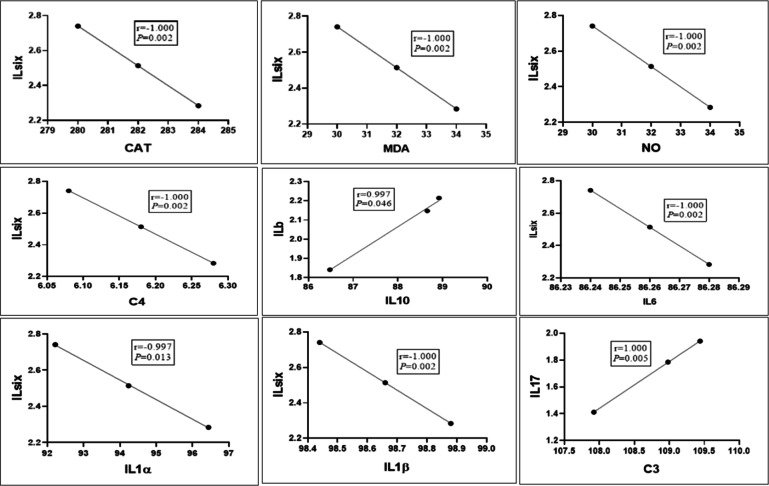
Correlations between gene expression patterns and serum profiles of immune and antioxidant markers

The mRNA levels of IL6 were negatively correlated with the serum levels of IL1α, IL1β, IL6, C4, CAT, NO, and MDA (*r* = −1, *p* = 0.002). Additionally, the mRNA levels of IL-1β were positively correlated with the serum IL-10 levels (*r* = 0.997, *p* = 0.04). The mRNA levels of IL17 were positively correlated with the serum levels of C3 (*r* = 1, *p* = 0.005) (Fig. 8).

## Discussion

Pneumonia is considered a major source of economic loss because of its frequent occurrence across all age groups and types of sheep, its high morbidity and mortality rates, and the associated slow growth and veterinary costs [59]. Desert-dwelling animals face stressors such as extreme summer heat and winter cold, increasing their vulnerability to microbial infections, especially respiratory diseases [60]. Our objective was to investigate the bacterial species and associated virulence gene profiles isolated from sheep exhibiting respiratory symptoms, as confirmed by PCR. This study also aimed to characterize the macroscopic and histological abnormalities observed in the affected lungs. Additionally, we sought to evaluate the potential role of genetic polymorphisms, oxidative stress biomarkers, and proinflammatory cytokines as diagnostic tools for pneumonia in Barki sheep.

Compared with healthy control sheep, the pneumonic sheep in this study presented harsh lung sounds upon auscultation, coughing, cyanotic mucous membranes, dyspnea, bilateral nasal discharge, elevated body temperature, and increased heart and respiratory rates. Our clinical findings align with those reported in sheep [12, 35, 61], buffalo calves [62], and calves [63–65]. The observed anorexia, depression, and dullness may result from muscular weakness due to intracellular potassium leakage, hyperkalaemia, and hypoglycemia [66]. Additionally, hyperthermia and pain responses can be attributed to infection and inflammation, stimulation of the hypothalamic thermoregulatory center, and the release of pyrogenic substances such as prostaglandins [67].

*Klebsiella pneumoniae* was isolated from 40% (60/150) of the pneumonic sheep in this study, as confirmed by PCR. This prevalence is higher than the 24.2% reported by [68] in Taif, KSA, but it is consistent with [69], who isolated *Klebsiella pneumoniae* from pneumonic sheep in Egypt by 36% and 48%, respectively. According to [70], the rate was 27.15%. On the other hand [71] and [72], from Austria and Brazil reported rates of 3.1% and 6.2%, respectively, whereas [41] from Iran reported a rate of 15.09%. Multiple virulence factors that aid in colonization and pathogenesis are present in *K. pneumoniae*. Particularly in immunocompromised animals, virulence factors make the organism more resilient to effective host defenses and make infection easier [73]. Both the *iutA* and the *fimbrial adhesin fimH* virulence genes were present in 39% and 68% of the *Klebsiella pneumoniae* strains, respectively. These findings support those of earlier research in Iran [74], but they are less extensive than those of [75] in Egypt, who isolated them from all samples and demonstrated that these virulence factors are critical for the pathogenicity of *K. pneumoniae*.

According to [76], *Pseudomonas aeruginosa* is an opportunistic pathogen that can cause a variety of infections in the gastrointestinal tract, respiratory system, ocular region, and other anatomical sites. The isolation rate of *Pseudomonas aeruginosa* in the current study was 18% (27/150). While [77] reported 20% in Egypt and [78] reported 21.8% in the USA, the average percentage results are in line with those previously reported by [27] at 15% in Egypt; however, the values are higher than those reported by [78] at 11.4% [79], at 10%, and [70] in Egypt at 9.1%. The *P. aeruginosa* isolation rate was 5.8%, according to [27] and [22] (16/27). The enterotoxin gene *(toxA)* is present in 59.2% of *Pseudomonas aeruginosa* isolates. This result was lower than that of [80] (69.4%) and [81] (76.6%) in Iran, but it was consistent with that previously reported by [27] (61%) in Egypt.

According to our data, *E. coli* was isolated from 30 out of 150 cases (20%). Ali & Abu-Zaid [69] reported that the incidence of *E. coli* in Egypt was 18%, whereas [82] reported a 20.78% isolation rate from respiratory-infected sheep in Diwaniya Governorate, Iraq. Pneumonia was also found in 19% of cases in Bikaner, Rajasthan, India [83], and in Bangladesh (20%), as a component of a mixed infection [84]. On the other hand [85], reported a 15.38% organism isolation rate from the lungs in Egypt, compared with 8% in [79] and 10.6% in [86]. In another study, *E. coli* strains of different serotypes were isolated at a rate of 21.8% [70], which was slightly higher than the rates reported by [87] and [88] in Egypt (72.7% and 69.7%, respectively).

Some strains of *E. coli* carry virulence genes that can cause intestinal or extraintestinal diseases, whereas others possess genes encoding toxins. The genetic diversity of *E. coli* strains offers valuable insights into their evolutionary history, relationships, and potential transmission risks to humans and animals [89]. The identification of pathogenic *E. coli* strains is facilitated by the presence of virulence factors. This information can be used to stop the spread of *E. coli* infections and to create new treatments [90].

*P. multocida* was found in 28.6% (43/150) of the isolates. These findings are somewhat at odds with those of [91], who reported that *P. multocida* was isolated at rates of 28.9% in Egypt. These rates, however, are greater than those reported by [79], who reported a 4% isolation rate; [92], who reported a 5% isolation rate in Egypt; and [93], who isolated *P. multocida* at rates of 11.4% (25/219) from healthy Iranian animals and 4.4% (5/114) from animals with pulmonary symptoms. In addition [94], reported isolation rates of 22% from Iran, 15.25% from pneumonic calves in Ethiopia [95], and 18.2% from Egypt [96], which were lower than those reported by [97] in Egypt (40%). Moreover [98], reported a prevalence of 65.62% from Iran, whereas [99] reported 62.50% from Egypt.

Following an initial trigger, such as long-distance transportation, poor ventilation, overcrowding, severe weather, or a weakened immune response, *Mannheimia haemolytica*, a commensal of the upper respiratory tract, can become pathogenic, leading to bronchopneumonia [100]. Our findings indicated that *M. haemolytica* was isolated from 31% (47/150) of the samples in this study, which is the overall isolation rate of this species. The findings of [101] in Ethiopia, which revealed a 34.21% isolation rate, closely match this outcome. In Ethiopia, *M. haemolytica* was isolated from sheep with a 28% isolation rate [102], which is somewhat consistent with the current investigation. The findings of [103] in India and [104] in Chhattisgarh, who reported isolation rates of 66.6% and 62%, respectively, in pneumonic lungs, are substantially higher than this rate. In contrast [105], reported a lower isolation rate of 21.87% in Ethiopia.

According to [106], *Mycoplasmas* species are frequently linked to respiratory illnesses in sheep and cause large financial losses. The current study revealed that *Mycoplasma spp*. were present in 44% (66/150) of the samples. In addition [72], reported *Mycoplasma spp*. in 38% of pneumonic sheep in Brazil; [107] reported a decrease of 7.4% in the lower respiratory tract of calves in Brazil. These results are consistent with those of [108] in Jordan, who found *Mycoplasma spp*. via PCR in 45.2% of examined sheep tracheal samples. However [108], reported that the prevalence in Swedish flocks was much higher, at 76%.

*Streptococcus* was isolated from the sick sheep analysed in 44 out of 150 cases (29.4%), which was consistent with the findings of another earlier study by [85], which reported a prevalence of 26.92%. Our data, on the other hand, were significantly higher than those of other studies; it was lower than the 55% reported by [109] in New Zealand and higher than the 3.9% reported by [82] in Iraq and [70] in Egypt, as well as the 15.5% mentioned by [72] in Brazil and the 14.8% reported by [110].

A positive correlation exists between larger areas and a higher prevalence of illness in warm climates, indicating that climate may influence respiratory health in these regions. Factors such as stress, humidity, dust, and overcrowding are likely to exacerbate these health issues [111]. The differences between the results of the present study and those of previous studies may stem from several factors, including variations in management practices, stressors, hygiene protocols, and the immune status of the infected animals [112].

Our results revealed that PCR-DNA sequencing of fragments of the *IL-1α* (472 bp), *IL-1B* (388 bp), *IL6* (526 bp), *TNF-α* (388 bp), *IL10* (526 bp), *LFA-1* (438 bp), *CR2* (381 bp), *IL17* (462 bp), *IL13* (376 bp), *DEFB123* (371 bp), *SCART1* (220 bp), *ICAM1* (399 bp), *SOD1* (387 bp), *CAT* (308 bp), *GPX1* (494 bp), *NOS* (540 bp), *HMOX1* (387 bp), and *NQO1* (377 bp) genes revealed nucleotide sequence variations in the form of SNPs between pneumonia-affected and healthy controls Barki ewes.

In our opinion, this is the first study to identify SNPs in antioxidant (SOD1, CAT, GPX1, NOS, HMOX1, and NQO1) and immunological (IL-1α, IL1B, IL6, TNF-α, IL10, LFA-1, CR2, IL17, IL13, DEFB123, SCART1, and ICAM1) genes as potential suspects for pneumonia resistance/susceptibility in Barki ewes. Interestingly, compared with the corresponding GenBank reference sequence, our results showed that the polymorphisms found in the investigated genes were reported here for the first time. Nonetheless, the candidate gene method was applied to evaluate ruminant susceptibility to pneumonia. The MHC-DRB1 exon 2 polymorphism, for example, was linked to *Mycoplasma ovipneumonia* resistance or susceptibility genotypes in sheep, according to [113]. The impact of TMEM154 gene polymorphisms on sheep vulnerability to ovine progressive pneumonia virus after natural exposure was investigated by [114]. In Baladi goats with pneumonia and healthy control group, PCR-DNA sequencing revealed SNPs linked to pneumonia susceptibility and resistance in immunological genes (SLC11A1, CD-14, CCL2, TLR1, TLR7, TLR8, TLR9, defensin, SP110, SPP1, BP1, A2M, ADORA3, CARD15, IRF3, and SCART1) [115].

The current study used real-time PCR to measure the levels of antioxidant and immunity-related mRNAs in both healthy controls and pneumonic Barki ewes. Our results revealed greater expression patterns of the IL-1α, IL-1B, IL6, TNF-α, LFA-1, CR2, IL17, IL13, DEFB123, SCART1, ICAM1, NOS, and HMOX1 genes in ewes with pneumonia than in healthy controls ewes. However, for the genes IL10, SOD1, CAT, GPX1, and NQO1, the opposite trend was observed. Our work is the first to use real-time PCR to determine immunological and mRNA levels in healthy controls and pneumonic ewes. Using genetic markers such as RFLP and SNPs, previous studies have investigated gene polymorphisms in domestic animals [113, 114]. Our study, however, was intended to address the drawbacks of earlier research by examining gene polymorphisms via gene expression and SNP genetic markers. Thus, the processes of gene regulation that have been studied in both healthy and pneumonia-affected lambs are well understood. The levels of immunological genes (SLC11A1, CD-14, CCL2, TLR1, TLR7, TLR8, TLR9, defensin, SP110, SPP1, BP1, A2M, ADORA3, CARD15, IRF3, and SCART1) vary in goats with and without pneumonia [115]. Complement genes and Toll-like receptors (TLRs) are linked to infectious pneumonia in sheep according to gene expression profiling [116].

In instances of inflammation, cytokines, including IL1α, IL-6, TNF-α, IL1B, IL17, and IL13, function as indirect indicators [117]. Among the primary proinflammatory cytokines that are involved in the immune response is TNF-α. TNF-α promotes the growth, development, and activity of numerous immune system cells, including B, T, NK (natural killer), and LAK (lymphokine-activated killer) cells, among others [118]. Additionally, a variety of additional cytokines are released in response to TNF-α [119]. TNF-α is encoded by a gene on chromosome BTA23q22 that has three introns and four exons [120]. Numerous mammalian cell types express TNF-α, with the highest levels observed in monocytes and macrophages. In these phagocytic cells, lipopolysaccharide (LPS) from bacterial cell walls triggers the synthesis of TNF-α. In LPS-stimulated macrophages, TNF-α gene expression increases threefold, mRNA levels increase approximately 100-fold, and protein secretion may increase by up to 10,000-fold [121]. According to [122], lipopolysaccharide-induced TNF-α factor (LITAF) is a new protein that binds to a crucial area of the TNF-α promoter and is thought to be involved in activating TNF-α expression after LPS induction. According to [123], β-defensins have been detected in vertebrates from a variety of tissues or cells, including blood plasma, urine, epithelial cells, neutrophils, and other leucocytes. During inflammation, defensins are released by neutrophils and monocytes (phagocytes) as microbicidal agents [124].

Together, these anti-inflammatory cytokines regulate the host inflammatory response to microbial antigens; interleukin-10 functions mainly as a feedback inhibitor of T-cell responses [125]. Leukocyte-specific integrins, known as beta 2 integrins, are expressed on the cell surface as heterodimers composed of the b subunit CD18 and the subunit CD11. Four distinct b2 integrins are produced by these subunit associations: CD11a/CD18 (LFA-1), CD11b/CD18 (Mac-1), CD11c/CD18 (CR4), and CD11d/CD18 [126]. Leukotoxin-beta 2-integrin interactions mediate the lethal effect of leukotoxin on ruminant leukocytes, according to previous research [127, 128]. Given its role in immune response regulation, CR2 is a viable candidate susceptibility gene for infection [129].

According to [130], the protein known as CD54 (cluster of differentiation 54), or intercellular adhesion molecule 1 (ICAM-1), is produced by the ICAM1 gene. This gene typically produces a cell surface glycoprotein on endothelial cells and immune system cells. T-cell-mediated host defense mechanisms and inflammatory processes are both mediated by ICAM-1 [130]. It is a costimulatory protein that activates MHC class II-restricted T cells on antigen-presenting cells and works in tandem with MHC class I to activate cytotoxic T cells on other cell types [130]. A protein that is present in only a specific subset of delta gamma T cells and is produced by the scavenger receptor family member expressed on T cells (SCART1) gene serves to recognize important pathogens [131]. However [132], via genome-wide association analysis, reported that the SCART1 gene was linked to foot health features in Holstein dairy cattle.

Antioxidants can scavenge or detoxify ROS, prevent their generation, or sequester transition metals that are the source of free radicals [133]. These systems constitute the body’s innate antioxidant defenses, such as glutathione peroxidase (GPx), catalase, and SOD, which are both enzymatic and nonenzymatic [134]. According to [135], type 2 nitric oxide synthase (NOS2) produces nitric oxide (NO), which is one of the primary ways that the host defends against infections. Through the 5-electron oxidation of the terminal guanidine-N2 of the amino acid L-arginine, constitutive endothelial or neural NO synthases or, at higher doses, inducible NO synthase (NOS2) produce nitric oxide.

LPS and other bacterial products, as well as cytokines, can trigger NOS2 expression [136]. Heme is broken down into equimolar amounts of carbon monoxide (CO), free iron, and biliverdin by the enzyme heme oxygenase (HO), which is the rate-limiting enzyme in the heme catabolic pathway [137]. It has been proposed to have a wide range of protective effects against many stressors, including anti-inflammatory, anti-apoptotic, anti-coagulation, anti-proliferative, vasodilative, and antioxidant effects. It is also referred to as a stress-responsive protein [138]. An antioxidant called NQO1 protects against the harmful oxidative and arylating effects of quinones [139]. NQO1’s detoxification mechanism for quinones is based on the direct two-electron reduction of quinones to hydroquinones, which eliminates electrophilic quinones and prevents the production of semiquinone radicals and reactive oxygen species through redox cycling processes [140].

The fact that injured tissues experience more free radical responses than healthy tissues may be the reason for the noticeable change in the expression patterns of immunological and antioxidant markers in lambs with pneumonia [141]. While the resistant genotypes dramatically increase the expression of IL-2 and IL-10, the susceptible genotypes stimulate inflammatory responses by increasing the expression of TNFa, IFNc, IL-4, IL-6, and IL-1b. Pneumonia also creates a variety of cellular immunological components that mediate and regulate the inflammatory response and immune function by binding to cell surface receptors [142]. Several investigations have shown that the relationships between these immune factors are complex. For example, a complex network structure is created by the interaction of these proteins, which affects the production of different immune globulins, complement proteins, and acute-phase reactive proteins [143]. Consequently, we hypothesize that the majority of the ewes in this study may have had infectious pneumonia. Additionally, there is solid evidence from our real-time PCR data that sheep with pneumonia experienced a significant inflammatory response.

According to the results of the present study, compared with the healthy group, the PG group presented a significant increase in the levels of proinflammatory cytokines. The immune system’s inflammatory response to an infection is triggered and intensified by these cytokines. By increasing the synthesis of humoral immune molecules, such as immunoglobulins (Igs), and innate immune molecules, such as acute-phase proteins, free radicals, complement factors, and matrix metalloproteinases can be produced. They are vital to the host’s defense. Our findings were similar to those reported by [14, 35] in sheep and [144, 145] cattle calves and [146] in cattle. On the other hand, anti-inflammatory cytokines are responsible for inhibiting the proinflammatory response to prevent excessive inflammation. Fernández et al. [147] reported that the decreased levels of anti-inflammatory cytokines in the PG likely worsened the condition by enabling the innate immune response to become more aggressive. This mismatch between pro- and anti-inflammatory signals exacerbates the situation and increases the severity of the inflammatory response during infection. Research by [35] revealed that acute pneumonic sheep had reduced IL-10 levels.

One characteristic of oxidative stress is the imbalance between antioxidants and free radicals, which was noted in this investigation. According to [148], free radicals are unstable molecules produced in trace amounts during cellular processes under normal physiological conditions. They try to stabilize by stealing electrons from molecules that are close by. By neutralizing these free radicals, antioxidants protect host cells and tissues from their damaging effects [149]. On the other hand, immune cells generate many free radicals when proinflammatory cytokines are activated during infection. These reactive molecules first attack and harm pathogen lipids, DNA, and carbohydrates, thereby assisting in their death [150]. Free radical buildup can eventually overwhelm the body’s antioxidant defenses. Free radicals then start attacking the host’s own cells, causing oxidative stress, a condition that aids in the pathogenesis of disease [149]. There have been prior reports of oxidative stress in sheep [59, 148] and calves [64, 65]. In addition to worsening tissue damage, oxidative stress is a major factor in disease progression.

The complex network of interacting β-globulins that attach to cell membranes or circulate freely in bodily fluids comprises the complement system, which is essential to innate lung immunity. As a first line of defense against infections, these complement proteins are generated by macrophages in response to proinflammatory cytokines [151]. Consistent with earlier findings, PG in this study significantly decreased the serum levels of the complement components C3 and C4 [152]. This decrease is most likely the result of hyperactivation of the complement cascade, which can be caused by bacterial lipopolysaccharides (LPS) via the alternative pathway or antigen‒antibody complexes (IgG and IgM) via the classical pathway [151].

Many immune effectors, such as membrane attack complexes (C5b--C9), opsonins (iC3b, C3b, and C4b), and chemoattractants (C3a, C4a, and C5a), are produced by the cleavage of C3 and C4 upon activation. According to [152], these molecules aid in the elimination of pathogens, stimulate neutrophil chemotaxis, encourage adhesion to endothelial cells, improve the respiratory burst in phagocytes, and modulate the acute inflammatory response by causing mast cell degranulation (releasing histamine). According to [151], the decreased levels of C3 and C4 found in this study are suggestive of severe lung damage, such as emphysema, airway dysfunction, gas trapping, and impaired lung elasticity. This depletion of complement proteins may be integral to the pathogenesis of bacterial pneumonia. Certain bacterial strains can undermine the complement system by producing toxins or surface proteins that interfere with its protective role in the lungs. As a result, complement proteins are consumed, increasing the bacterial load and amplifying the production of proinflammatory cytokines such as IL-1β and TNF-α, which further exacerbates lung inflammation [152].

The activation of humoral immunity in the PG is also highlighted by the observed increase in immunoglobulins (γ-globulins) in the PG. This increase aligns with earlier research [35]. Igs that B cells generate in response to cytokines promote inflammation. By encouraging microbial antigen phagocytosis, neutralizing bacterial toxins, competing with pathogens for host cell attachment, precipitating microorganisms, and initiating the complement cascade, they strengthen the immune response. Together, these processes strengthen the body’s defenses against infection and highlight the role that humoral immunity plays in preventing bacterial pneumonia [152].

With respect to the diagnostic and prognostic value of cytokines and oxidative stress markers, they had high values of sensitivity, specificity, PPV, NPV, and accuracy rate at AUC = 1 and moderate LRs. This result partially agreed with previous data suggesting that proinflammatory cytokines and oxidative stress indicators are sensitive markers for pneumonia in sheep [14, 35, 153], goats [154] and cattle calves [148], but this result disagrees with these studies in terms of marker preference, as it prioritizes proinflammatory cytokines among oxidative stress markers (due to their percentage increase, especially IL-1α) and excludes NO (due to its low specificity and LR). Several factors may result in this disagreement, such as animal age, nutrition and breed, disease stage, etiology, and method of marker estimation.

In the present study, the anatomic lung lesions in the pneumonic sheep matched the macroscopic pathological abnormalities in the lungs, which presented much more severe inflammatory lesions characterized by extensive red and gray hepatization and congestion and a marbling appearance [155]. Lung histopathological analysis revealed giant alveoli, alveolar emphysema, granulomas, serous pneumonia, and congestion, which supported earlier findings [61, 156].

## Conclusion

This study revealed a diverse range of bacterial pathogens associated with respiratory illnesses in sheep flocks in Egypt, underscoring the importance of their identification and isolation. Our findings highlight the importance of SNPs in immune and antioxidant genes as genetic markers influencing pneumonia in healthy and pneumonic sheep. The genetic variability in these genes could serve as proxy biomarkers for pneumonia in Barki sheep. Additionally, the distinct expression patterns of immune and antioxidant genes in healthy and pneumonic Barki sheep may provide valuable insights for monitoring their health status. These findings offer a promising opportunity to mitigate pneumonia through selective breeding based on genetic markers linked to natural resistance. Finally, sheep pneumonia is characterized by alterations in the biochemical profile of immunological and antioxidant markers, triggering both innate and humoral immune responses.

## Data Availability

No datasets were generated or analysed during the current study.

## Abbreviations

IL-1α: Interleukin 1 alpha
IL-1β: Interleukin 1 beta
IL-6: Interleukin 6
TNF-α: Tumor necrosis factor-alpha
IL10: Interleukin 10
LFA-1: Lymphocyte function-associated antigen 1
CR2: Complement C3d receptor 2
IL-17: Interleukin 17
IL-13: Interleukin 13
DEFB123: β-defensin
SCART1: Scavenger Receptor Family Member Expressed on T Cells 1
ICAM1: Intercellular adhesion molecule 1
SOD1: Superoxide dismutase 1
CAT: Catalase
GPX1: Glutathione peroxidase 1
NOS: Nitric oxide synthetase
HMOX1: Heme oxygenase 1
NQO1: NAD(P)H quinone dehydrogenase 1
C3: Complement 3
C4: Complement 4
IgG: Immunoglobulin G
IgM: Immunoglobulin M
IgA: Immunoglobulin A
MDA: Malondialdehyde
NO: Nitric oxide
CAT: Catalase
GPx: Glutathione peroxidase
GR: Glutathione reductase
